# A Rare Case of Angioimmunoblastic T-Cell Lymphoma with Epstein-Barr Virus-Negative Reed-Sternberg-Like B-Cells, Chylous Ascites, and Chylothorax

**DOI:** 10.1155/2017/1279525

**Published:** 2017-04-12

**Authors:** Mathijs Willemsen, Arne W. J. H. Dielis, Iryna V. Samarska, Ad Koster, Arienne M. van Marion

**Affiliations:** ^1^Department of Internal Medicine, VieCuri Medical Centre, 5912 BL Venlo, Netherlands; ^2^Department of Pathology, VieCuri Medical Centre, 5912 BL Venlo, Netherlands

## Abstract

Angioimmunoblastic T-cell lymphoma is a rare non-Hodgkin lymphoma with dismal prognosis. The median age of presentation ranges from 62 to 69 years with generalized lymphadenopathy, B symptoms, and hepatosplenomegaly as the most prevalent symptoms. The combination of B-cell and T-cell proliferations is common in AITL and the B-cell component may resemble Reed-Sternberg-like B-cells. Epstein-Barr virus is estimated to be present in 80–95% of AITL biopsies. Only a handful of EBV-negative AITL cases with EBV-negative RS-like B-cells have been reported over the last decade. We present a rare case of EBV-negative AITL with chylous ascites and chylothorax. Microscopic and immunohistochemical analysis revealed the presence of EBV-negative Reed-Sternberg-like B-cells in the tumor.

## 1. Introduction 

Peripheral T-cell lymphoma (PTCL) defines a group of rare non-Hodgkin lymphomas (NHL) characterized by an aggressive clinical course and dismal prognosis. Angioimmunoblastic T-cell lymphoma (AITL) accounts for 15–20% of all PTCL cases worldwide with the highest incidence rate in Europe (28,7%) [1]. The 5-year overall survival rate ranges from 25% to 41% with no survival improvement for AITL patients over the last two decades [2]. The median age of presentation ranges from 62 to 69 years with generalized lymphadenopathy, B symptoms (e.g., fever, night sweat, and weight loss) and hepatosplenomegaly as the most prevalent symptoms. Skin rash occurs in 20–60%, eosinophilia in 30–40%, and ascites in 20–40% of all cases [1, 3, 4]. Additional laboratory findings often include anemia, elevated lactate dehydrogenase, hypergammaglobulinemia, and the presence of autoantibodies and immune complexes (e.g., positive Coombs test, cold agglutinins, cryoglobulins, rheumatoid factor, and antismooth muscle antibodies) [1, 3, 4]. Over 80% of patients present with advanced disease at the time of diagnosis (Ann Harbor stages III-IV) [1, 2].

Epstein-Barr virus (EBV) is estimated to be present in 80–95% of AITL biopsies. Although EBV is not believed to play a primary role in AITL oncogenesis, it potentially contributes to establishing a clonal or oligoclonal B-cell population, including Reed-Sternberg-like B-cells (RS-like B-cells) in combination with an expanding T-cell population with tumorous behavior [5–7]. Reports of clonal and oligoclonal B-cell populations in EBV-negative AITL suggest that other mechanisms than EBV infection contribute to expansion of the B-cell population in AITL [6, 8].

## 2. Case Report 

A 76-year-old Caucasian male with a history of coronary artery disease, Barrett's esophagus, and two recent episodes of allergic vasculitis presented with generalized erythematosquamous plaques, bilateral pitting edema of the lower extremities, and weight loss.

Physical examination revealed cervical lymphadenopathy, generalized rhonchi, and bilateral pitting edema of the lower extremities. Oxygen saturation was 92% while breathing room air. Other vital signs were normal. Exploratory laboratory tests showed elevated lactate dehydrogenase (445 U/L), severe renal dysfunction (BUN 31.7 mmol/L, creatinine 236 *µ*mol/L), microcytic anemia (Hb 6.1 mmol/L, MCV 77 fl), mild inflammation (CRP 88 mg/L), thrombocytopenia (124 · 10^9^/L), and normal leukocyte count (9.0 · 10^9^/L) with mild eosinophilia (990/*μ*L). Chest radiography showed mild bilateral pleural effusion. Biochemical and microscopic urinalysis were normal. Skin biopsy showed signs of common dermatitis without the presence of atypical cells or infiltrating eosinophils.

On the ward, fluid resuscitation and discontinuation of antihypertensive drugs almost completely restored renal function (BUN 7.5 mmol/L, creatinine 107 *μ*mol/L) within seven days. Nevertheless, additional laboratory tests showed progressive hematologic abnormalities revealing normal leukocyte count (7.4 · 10^9^/L) with eosinophilia (1332/*μ*L), thrombocytopenia (45 · 10^9^/L), and microcytic anemia (Hb 5.0 mmol/L, MCV 76 fl). During the following days the patient's condition deteriorated with complete loss of appetite, increasing pitting edema of the lower extremities, need for oxygen supplementation, transfusion of blood products, and fever (39°C). Comprehensive diagnostic work-up was unremarkable (Table 1). Suspecting a hematologic malignancy, bone marrow biopsy was performed and a PET/CT and flow cytometry were ordered. Unfortunately, the patient required cardiopulmonary resuscitation after aspiration and died sixteen days after admission. Family gave informed consent to perform an autopsy.

Autopsy showed signs of aspiration of stomach contents. The pleural cavity contained 600 mL of milky fluid. Microscopic examination of the lungs showed massive peribronchial infiltration of lymphocytes, neutrophils, and plasma cells with features of aspiration on the background.

**Figure 1:**
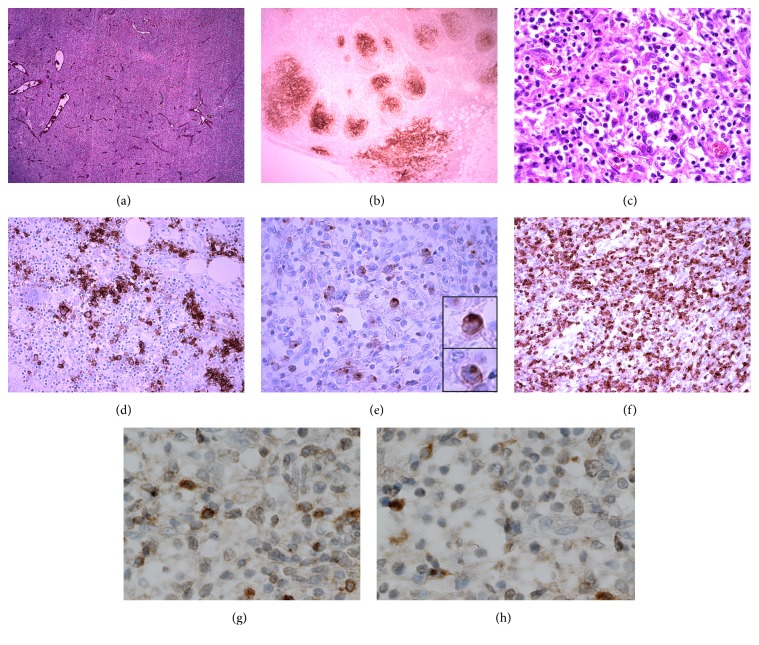
The histology and immunohistochemistry of a lymph node. (a) Gross examination of the lymph node architecture shows prominent vessels, suggesting the formation of the arborizing high endothelial venules. (b) The expanded CD21-positive follicular dendritic cell meshworks. (c) Hematoxylin and eosin stain showing polymorphous background infiltrate composed of medium sized atypical lymphocytes and enlarged pleomorphic cells with unilobated nuclei and prominent nucleoli, resembling RS-like B-cells. (d) The RS-like B-cells have less prominent CD20 expression in comparison to the B-lymphocytes present in the polymorphous infiltrate. (e) The RS-like B-cells are CD30-positive with characteristic paranuclear dot-like staining. (f) The lymphoid population consists of the CD2-positive atypical T-lymphocytes that often form rosettes around RS-like B-cells. (g) and (h) Atypical T-lymphocytes are CD10- and BCL6-positive.

The ileum, jejunum, and colon had a patchy milky surface with dilated serosal lymphatic vessels. The peritoneal cavity contained 1250 mL of milky fluid. Biochemical analysis of pleural effusion and ascites showed an elevated level of triglycerides (2.6 mmol/L), indicative of chylothorax and chylous ascites. Significant lymphadenopathy was seen in the cervical, thoracic, and abdominal compartments. Microscopic examination revealed effacement of the normal nodular architecture with predominantly small-sized lymphoid cells with cleaved nuclei and scant cytoplasm distributed among a polymorphous background infiltrate including small lymphocytes, eosinophils, plasma cells, and histiocytes (Figure 1(c)). Enlarged cells resembling Hodgkin-like cells or RS-like B-cells with abundant pale cytoplasm and unilobated and enlarged nuclei with prominent eosinophilic nucleoli were noticed (Figure 1(c)). Evident proliferation of arborizing high endothelial venules was observed (Figure 1(a)).

**Figure 2:**
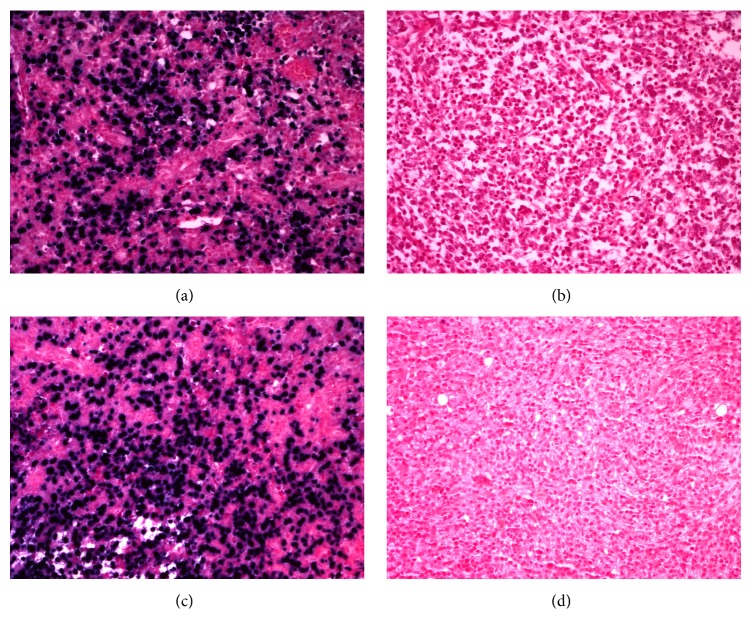
EBER-ISH of lymph node and bone marrow. (a) and (c) Positive controls of lymph node and in bone marrow, respectively. (b) and (d) Negative EBER-ISH in the lymph node and bone marrow of the current patient, respectively.

Immunohistological analyses demonstrated a diffuse increase in CD3-positive T-lymphocytes that were also CD2-, CD4-, and CD5-positive (Figure 1(f)). The number of CD4-positive T-lymphocytes was markedly increased compared to CD8-positive T-lymphocytes. Atypical T-lymphocytes were CD10- and B-cell lymphoma 6- (BCL6-) positive, indicative of T-follicular helper (TFH) phenotype (Figures 1(g) and 1(h)). Sporadic CD20- and PAX-5-positive B-lymphocytes were present in the residual follicles. The RS-like B-cells were CD20- and CD30-positive (Figures 1(d) and 1(e)). The Epstein-Barr encoding region in situ hybridization (EBER-ISH) was negative (Figure 2). The CD21 stain revealed the preexistent follicular dendritic networks (“burn out” lymphoid follicles); however an expansion of those networks with the formation of enlarged nodular structures was also seen (Figure 1(b)). Stains for CD56, CD15, and ALK-1 were negative.

**Figure 3:**
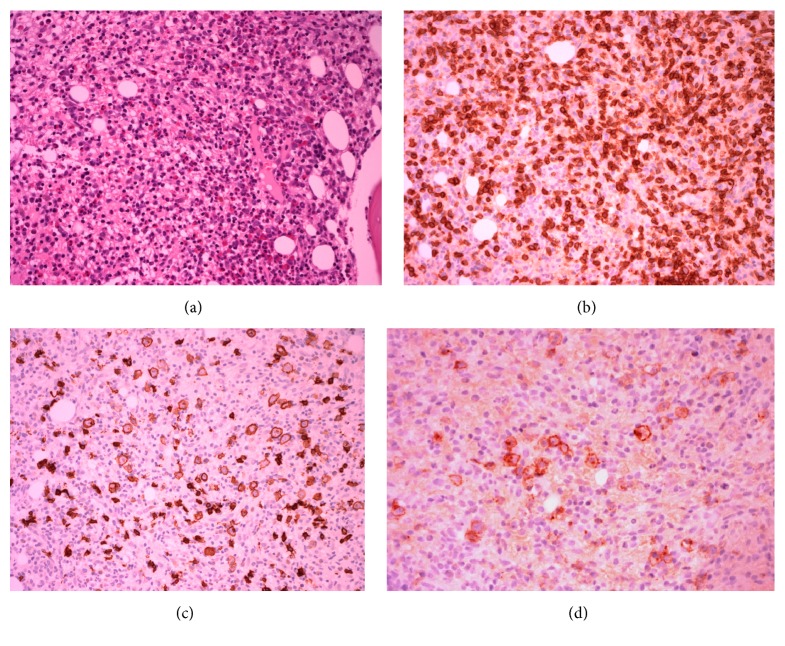
Histology and immunohistochemistry of bone marrow. (a) Hypercellular bone marrow with prominent lymphoid infiltration. (b) Atypical CD3-positive T-lymphocytes. (c) Enlarged pleomorphic cells with unilobated nuclei and prominent nucleoli, resembling RS-like B-cells. These cells show less pronounced expression of CD20 compared to normal B-lymphocytes (a CD20-positive small cell population in the background). (d) The RS-like B-cells are CD30-positive with characteristic paranuclear dot-like staining.

The premortal bone marrow biopsy showed hypercellularity with marked interstitial infiltration of small lymphocytes with cleaved nuclei and scant cytoplasm (Figure 3(a)). These lymphocytes were CD3-positive and were organized in paratrabecular clusters together with a high number of CD68-positive histiocytes and eosinophils (Figure 3(b)). The CD20- and CD30-positive RS-like B-cells were distributed within this population (Figures 3(c) and 3(d)). EBER-ISH was negative (Figure 2).

Based on the histological features of the enlarged lymph node, with nodular expanding dendritic networks and high endothelial venules, combined with infiltrating CD4-positive atypical small T-lymphocytes with TFH phenotype, scattered CD30- and CD20-positive RS-like B-cells, and clinical signs of systemic lymphadenopathy with chylothorax and chylous ascites, the patient was diagnosed with AITL. Based on the autopsy findings, the bilateral aspiration bronchopneumonia was suggested as the cause of death. The diagnosis was confirmed in an expert panel of hematopathologists.

## 3. Discussion 

The presence of chylothorax and chylous ascites in AITL is rare. A literature search yielded only one English written case reporting on the presence of chylothorax in AITL [9]. Chylothorax is a complication following thoracic duct damage or obstruction by systemic lymphadenopathy. Malignancy is the most common cause of nontraumatic thoracic duct obstruction. In about 70% of cases lymphoma is found, with the majority being NHL [10]. Chylous ascites is a form of ascites most commonly caused by recent surgery or trauma. A recent systematic review revealed that 25% of adult cases of nontraumatic chylous ascites are attributable to malignancy. In approximately one-third of these cases NHL is found to be the underlying condition [11]. Patients presenting with chylothorax and/or chylous ascites without recent surgery or trauma should be readily evaluated for the presence of malignancy, especially lymphoma.

The current report discusses the case of a patient with EBER-ISH-negative AITL with systemic lymphadenopathy. Postmortal examination of an enlarged lymph node showed typical features of AITL, including effacement of nodal architecture with “burn out” lymphoid follicles (pattern II), proliferation of arborizing high endothelial venules, and the infiltration of CD4-positive atypical small T-lymphocytes with TFH phenotype [7]. The 2016 revision of the World Health Organization classification of lymphoid neoplasms introduces the umbrella term “nodal T-cell lymphomas with T-follicular helper phenotype,” which includes AITL, follicular T-cell lymphoma, and nodal PTCL with a TFH phenotype. This decision is based on overlapping clinicopathological findings and similar mutational landscape among these lymphomas [12, 13]. Emerging evidence suggests that TFH cell-derived lymphomas are part of a single spectrum of disease, with AITL being considered as “prototypic” TFH cell neoplasm [12]. Additionally, we identified scattered CD30- and CD20-positive and CD15-negative RS-like B-cells in both lymph nodes and bone marrow. Identification of RS-like B-cells in subtypes of PTCL has led to a diagnostic dilemma as classical Hodgkin lymphomas are also characterized by a polymorphous background infiltrate. However, for AITL, identification of typical immunomorphological features such as proliferation of arborizing high endothelial venules and infiltration of atypical T-lymphocytes, negative PAX-5 immunostaining, and expression of CD3 and CD4 on a subset of RS-like B-cells can be used to avoid misdiagnosis [7, 14].

In the present case, EBER-ISH was negative. Epstein-Barr virus (EBV) is estimated to be present in 80–95% of AITL biopsies. Although EBV is not believed to play a primary role in AITL oncogenesis, it potentially contributes to establishing a clonal or oligoclonal B-cell population. EBV infection promotes survival of B-cells which subsequently undergo clonal expansion. Proliferation of B-cells may ultimately lead to development of a secondary malignant B-cell lymphoma [6, 7]. Recently, three cases of AITL with EBER-ISH-negative RS-like B-cells have been described [8]. EBER-ISH-negative B-cell lymphomas have also been described in the context of PTCL [5]. These results indicate that EBV infection is not the only mechanism contributing to expansion of B-cell population in AITL. As in our case, neoplastic T-cells with TFH phenotype were found to form rosettes around the EBER-ISH-negative RS-like B-cells [8]. TFH cells are a subset of CD4-positive T-cells expressing BCL6, CD10, programmed cell death 1 (PD-1), inducible T-cell costimulator, interleukin-21, and chemokine receptor CXCR5. TFH cells primarily reside in germinal centers where they interact with activated B-cells to enable the production of high affinity isotype switched antibodies and maintain humoral memory [15]. It has been hypothesized that the TFH phenotype of neoplastic T-cells is responsible for the clonal expansion of B-cell population in EBER-ISH-negative AITL cases. Additionally, the high expression of PD-1 and rosetting around RS-like B-cells potentially creates an immunological barrier which protects the expanding B-cell population [5, 8].

Recently, researchers reported on a Japanese cohort of 30 PTCL cases with RS-like B-cells, including 12 AITL cases [16]. Only two of the AITL cases had EBER-ISH-negative RS-like B-cells, underlining the rarity of this phenomena. The researchers showed that EBER-ISH status of RS-like B-cells in PTCL does not influence survival or any other clinical parameter. Interestingly, B-cell-associated laboratory findings were not taken into account [16]. Despite the high incidence of B-cell-associated laboratory findings in AITL our patient tested negative (Table 1). One could hypothesize that EBER-ISH-negative and EBER-ISH-positive AITL cases present with distinct profiles of B-cell-associated laboratory findings. This may explain the absence of B-cell-associated findings in the current case.

In conclusion, we present a rare case of advanced stage, EBER-ISH-negative AITL with chylothorax, and chylous ascites. We also identified scattered CD30- and CD20-positive and CD15- and EBER-ISH-negative RS-like B-cells. Care must be taken to avoid misdiagnosis of classical Hodgkin lymphoma. Only a handful of AITL cases with EBER-ISH-negative RS-like B-cells have been reported in the literature and clinical implications are yet to be determined. Future research should be conducted to elucidate the precise nature of the interaction between neoplastic T-cells in AITL and B-cells which lead to the emergence of EBER-ISH-negative RS-like B-cells. Hopefully, this will lead to a more in-depth knowledge of AITL oncogenesis and uncover targeted therapies for EBER-ISH-negative AITL.

## Figures and Tables

**Table 1:** Differential diagnostic considerations and results of corresponding diagnostic tests.

Diagnosis	Diagnostic test(s)
Eosinophilic granulomatosis with polyangiitis	No ANCAs and anti-MPO antibodies

Granulomatosis with polyangiitis	No ANCAs and anti-PR3 antibodies

Systemic lupus erythematosus	No ANAs and ENAs Normal complement C3 (1.4 g/L) and C4 (0.29 g/L)

Antiphospholipid syndrome	Normal IgM and IgG beta-2 glycoprotein (ratio 0.08 and 0.17) and IgM and IgG cardiolipin antibodies (ratios 0.11 and 0.35)

Autoimmune hemolytic anemia	Direct Coombs test negative

Rheumatoid arthritis	Normal RF (24 UI/mL) and ACPAs (0.0 IU/mL)

Cryoglobulinemia	No cryoglobulins

HIV/AIDS	HIV screening negative

Prostate cancer	Low PSA (0.7 *µ*g/L)

Chronic myeloid leukemia (CML)	No BCR-ABL fusion gene

Non-CML myeloproliferative disorders	No JAK2 V617F, JAK2 exon 12, CALR exon 9 or MPL exon 10 mutation

Chronic eosinophilic leukemia Hypereosinophilic syndrome	No amplification, deletion or rearrangement of FIP1L1- CHIC2-PDGFRA (4q12), PDGFRB (5q32-33) or FGFR1 (8p12) genes

Multiple myeloma Waldenström's macroglobulinemia	Normal serum plasma electrophoresis (albumin 45 g/L, alpha-1 globulin 1 g/L, alpha-2 globulin 10 g/L, beta globulin 8 g/L, gamma globulin 9 g/L)

Upper gastrointestinal malignancy	No dysplasia on endoscopic biopsies

ANCAs: anti-neutrophil cytoplasmic antibodies, MPO: myeloperoxidase, PR3: proteinase 3, ANAs: antinuclear antibodies, ENAs: extractable nuclear antigens (nRNP/Sm, Sm, SSA60, Ro-52, SSB, Scl-70, CENP-B, Jo-1, PM-scl and ribosomal P protein), RF: rheumatoid factor, ACPAs: antibodies to citrullinated protein antigens, HIV: human immunodeficiency virus, AIDS: acquired immune deficiency syndrome, PSA: prostate-specific antigen.
